# Farm animal coronaviruses: the solution is in vaccines

**DOI:** 10.1080/01652176.2025.2556494

**Published:** 2025-09-17

**Authors:** Olga Kondakova, Anna Tsybina, Ekaterina Evtushenko, Ekaterina Ryabchevskaya, Dmitriy Granovskiy, Angelina Kovalenko, Marina Arkhipenko, Nikolai Nikitin, Olga Karpova

**Affiliations:** Department of Virology, Lomonosov Moscow State University, Moscow, Russia

**Keywords:** Coronaviruses, genotypes, farm animals, vaccines, zoonotic potential

## Abstract

RNA-containing coronaviruses are widespread in nature and can infect a number of vertebrates. Animals are potential sources of human coronaviruses diseases, and interspecies infection by animal coronaviruses has been recorded several times. Such a transmission may have caused the COVID-19 pandemic. The study and control of the spread of farm animals’ coronavirus infections is very important, due to the constant close contact between humans and farm animals. Vaccination remains the key to preventing animal diseases and limiting the further spread and transmission of coronavirus infections among poultry and livestock. This review considers coronavirus infections in farm animals, which pose a serious challenge to animal husbandry, and their zoonotic potential and epidemiological features. The review also discusses current vaccines and their limitations, as well as the latest developments and trends in veterinary vaccines aimed at preventing coronavirus infections in poultry and livestock.

## 1. Introduction

The *Coronaviridae* family (order *Nidovirales*) comprises RNA-containing viruses that are widely distributed in nature and capable of infecting a number of species of vertebrates. Coronaviruses (CoVs) that infect mammals and birds belong to the *Orthocoronavirinae* subfamily, which has four genera: *Alphacoronavirus*, *Betacoronavirus*, *Gammacoronavirus* and *Deltacoronavirus* (Woo et al. 2023). Over the past two decades, a significant number of new virus species and strains have been identified within this subfamily. Moreover, three new deadly zoonotic coronavirus infections have emerged in the human population: SARS-CoV, MERS-CoV and SARS-CoV-2. Mammalian CoVs primarily belong to the *Alpha-* and *Betacoronavirus* genera. Specifically, the *Alphacoronavirus* genus includes seasonal human CoVs (HCoV-229E and HCoV-NL63), as well as swine CoVs, such as transmissible gastroenteritis virus (TGEV) and porcine epidemic diarrhea virus (PEDV). The *Betacoronavirus* genus encompasses five viruses that infect humans: HCoV-OC43, HCoV-HKU1, SARS-CoV, MERS-CoV and SARS-CoV-2, as well as bovine coronavirus (BCoV). In contrast, gamma- and deltacoronaviruses mainly infect birds, with exceptions such as porcine deltacoronavirus (PDCoV) and marine mammal CoVs belonging to the *Gammacoronavirus* genus. Since the discovery of the first coronavirus, which was infectious bronchitis virus (IBV) in chickens, in the 1930s, members of the *Orthocoronavirinae* subfamily have been identified in a variety of farm animals, including pigs and cattle (Table 1). These viruses can cause large-scale disease outbreaks and pose significant economic challenges to the livestock and poultry industry.

**Table 1.** Farm animal coronaviruses and their licensed vaccines.

Animal	Coronavirus (genus)	Year of discovery	Symptoms and severity of the disease	Spread of disease	Licensed vaccines
Chickens	IBV (*Gammacoronavirus*)	1933 (USA)	Respiratory and intestinal disorders, kidney damage. Severe forms of the disease in chickens	Global	Live attenuated (Russia, China, India, Japan, South Korea, Europe, Brazil, Canada, Australia, USA) Inactivated (Russia, Europe, China, Japan, Brazil, Canada, USA) Turkey Herpesvirus Vectored (Europe, USA, Latin America, China, Middle East and Africa)
Cattle	BcoV (*Betacoronavirus*)	1972 (USA)	Neonatal calf diarrhea, winter dysentery, respiratory disorders Moderate and high severity of the disease	Global	Live attenuated (USA, Canada, Europe) Inactivated (USA, Europe, Russia)
Pigs	TGEV (*Alphacoronavirus*)	1946 (USA)	Severe diarrhea with dehydration in animals of all ages. High mortality in newborn piglets (up to 100%)	Global	Live attenuated (Europe, China, Japan, USA), Inactivated (Russia, China)
	PEDV (*Alphacoronavirus*)	1971 (Great Britain)	Severe diarrhea with dehydration in animals of all ages. High mortality in newborn piglets (up to 100%)	Global	Live attenuated (Japan, Philippines, China) Inactivated (Canada, South Korea, USA) Recombinant alphavirus-based (USA)
	PDCoV (*Deltacoronavirus*)	2012 (China)	Severe diarrhea with dehydration in animals of all ages. High mortality in newborn piglets (up to 100%)	Global	No
	SADS-CoV (*Alphacoronavirus*)	2017 (China)	Severe diarrhea with dehydration in animals of all ages. High mortality in newborn piglets (up to 100%)	China	No
	PHEV (*Betacoronavirus*)	1962 (Canada)	Vomiting and wasting syndrome, neurological and/or respiratory symptoms. Severe course of the disease and high mortality (up to 100%) in neonatal piglets	Global	No
	PRCV (*Alphacoronavirus*)	1983 (Belgium)	Subclinical form or mild respiratory symptoms	Global	No

Abbreviation: IBV - Infectious bronchitis virus; BCoV - Bovine coronavirus*;* TGEV - Transmissible gastroenteritis virus; PEDV - Porcine epidemic diarrhea virus*;* PDCoV - Porcine deltacoronavirus; SADS-CoV - Swine acute diarrhea syndrome coronavirus*;* PHEV - Porcine hemagglutinating encephalomyelitis virus; PRCV - Porcine respiratory coronavirus.

Before the emergence of SARS-CoV, in 2002–2003, coronavirus research primarily focused on veterinary aspects, including the development and application of veterinary vaccines (Table 1). However, interest in this virus family surged following the SARS-CoV outbreak, as it became evident that coronavirus infections could pose a serious threat to humans, even leading to fatalities. The genetic diversity of CoV strains, their high mutation and recombination rates, and human-animal interactions create potential opportunities for cross-species transmission to humans. In the last decade, two new swine CoV species have been identified: swine acute diarrhea syndrome coronavirus (SADS-CoV) and PDCoV, with the latter already confirmed to have zoonotic potential (Lednicky et al. 2021).

Vaccination plays a crucial role in preventing animal diseases and curbing further CoV transmission among humans and animals. However, commercially licensed vaccines that have been used for decades to protect farm animals against CoVs have been demonstrated to have challenges similar to those encountered with SARS-CoV-2 vaccines. These vaccines significantly reduce clinical symptoms and the viral load in biological samples but still fail to completely prevent viral spread within animal populations (Saif 2004; Hassebroek and Meng 2023). Controlling the spread of CoV requires updated vaccine formulations and new rounds of immunisation. Nevertheless, the vast experience gained from the development and licensing of SARS-CoV-2 vaccines has spurred the creation of next-generation vaccines targeting economically significant coronaviruses in the agricultural sector (Table 2).

**Table 2.** Vaccines currently in development for the prevention of coronavirus infection in cattle, swine, and poultry (2020–2025).

Virus	Type of vaccine	Antigen(s)	Adjuvant	Route of administration	Developer	Development stage	References
Coronavirus infections in cattle							
BCoV	Live attenuated	KBR-1-p120 (subgroup GIIa)	No	PO	Virus Disease Division, Animal and Plant Quarantine Agency (South Korea)	Pathogenic reversion test in cows	Park et al. 2024
	Subunit	Epitopes of HE, S, M, N proteins	–	–	Agricultural University Urumqi (China)	In silico*	Jiang et al. 2024
	Subunit	Epitopes of HE, N, S, E, M proteins	–	–	Long Island University, Brooklyn (USA	In silico*	Duraisamy et al. 2024
	Subunit	Epitopes of HE and S proteins	–	–	University of Bahri (Sudan)	In silico*	Awadelkareem and Hamdoun 2024
	VLP	E, M, N, S, HE	MF59 + CPG55.2	IM	Southwest Minzu University, Chengdu (China)	Immunogenicity in mice and cattle and virus-neutralizing activity of mice sera *in vitro*	Yu et al. 2024a
	Viral vector (Ad)	M, S	No	IM	University of Parma (Italy)	Immunogenicity in sheep	Pratelli et al. 2024
Coronavirus infections in poultry							
IBV	Recombinant inactivated virus-based	H120-CSL	Oil emulsion	SC or IN	South China Agricultural University (China)	Safety, cross-protection of chicken sera and protective efficacy in chicken	Huang et al. 2025
	Chimeric VLP	S	No	Inoculation in the breast	University of Pretoria, Pretoria (South Africa)	Immunogenicity and protective efficacy in chicken	Sepotokele et al. 2023b
	Bacterial vector *(Salmonella spp)*	S1	Emulsigen^®^-P	PO	Yangzhou University China (China); University of Florida (USA)	Immunogenicity and protective efficacy in chicken	Liu et al. 2024
	DNA	The consensus sequence of the S protein ectodomain	No	IM	Sichuan University (China)	Immunogenicity in mice, virus-neutralizing activity of mice sera *in vitro* and immunogenicity and protective efficacy in chicken	Zuo et al. 2021
	Viral vector (LSDV)	S and N proteins consensus sequences	–	IM	University of Cape Town, Cape Town (South Africa)	Immunogenicity in mice	Chineka et al. 2025
Coronavirus infections in swine							
TGEV	Subunit	Conserved S protein epitopes	–	–	Hunan University (China)	In silico*	Bai et al. 2024
	Subunit	S protein epitopes	–	–	Shihezi University (China)	In silico*	Li et al. 2025
PEDV	Live attenuated	17GXCZ-1ORF3d-P120 (genogroup GIIb)	No	IM	Guangxi University, Nanning (China)	Immunogenicity and protective efficacy in pigs	Lu et al. 2024a
	Inactivated	ShXXY2-2023 (genogroup GIIb)	201	IM	Sun Yat-sen University, Guangzhou (China)	Immunogenicity and protective efficacy in pigs and virus-neutralizing activity of swine sera *in vitro*	Hu et al. 2025
	Subunit	S protein trimer	Montanide IMS 1313	IM	Kyushu University Graduate School of Bioresource and Bioenvironmental Sciences (Japan)	Immunogenicity in mice and virus-neutralizing activity of mice sera *in vitro*	Masuda et al. 2021
	Subunit	S protein trimer, S1 subunit, COE	M103 or M401	IM	Institute of Veterinary Medicine (China)	Immunogenicity in mice and pigs, virus-neutralizing activity of mice sera *in vitro* and protective efficacy in pigs	Guo et al. 2024
	Subunit	S protein trimer (genogroups GIIa and GIIb)	M103	IM	Hebei Agricultural University (China)	Immunogenicity and protective efficacy in pigs and virus-neutralizing activity of swine sera *in vitro*	Song et al. 2024b
	Subunit	S1 subunit trimer, COE trimer, RBD trimer	IAS 201	IM	Lanzhou University and others (China)	Immunogenicity in mice and pigs, virus-neutralizing activity of mice and swine sera *in vitro* and protective efficacy in pigs	Li et al. 2024d
	Subunit	*S protein epitopes	–	–	Shanghai Medicilon Inc., Shihezi University, Xinjiang Western Animal Husbandry Co., Ltd (China)	In silico*	Li et al. 2024e
	Subunit	Spike ectodomain	MONTANIDE^™^ ISA 201 VG	IM	Guangdong Enterprise Key Laboratory for Animal Health and Environmental Control, Wen’s Foodstuff Group Co. Ltd, Yunfu (China)	Immunogenicity and protective efficacy in pigs and virus-neutralizing activity of swine sera *in vitro*	Liu et al. 2024
	VLP	S, M and E	Freund’s adjuvant + CCL25 or CCL28	IM	National Taiwan University, Chung Yuan Christian University, China Medical University Taiwan (Taiwan)	Immunogenicity and protective efficacy in pigs and virus-neutralizing activity of swine sera *in vitro*	Hsu et al. 2020
	VLP	S, M and N	Alum adjuvant	IM	Chungnam National University (South Korea)	Immunogenicity in mice and virus-neutralizing activity of mice sera *in vitro*	Kim et al. 2021
	Bacterial vector *(L. johnsonii)*	COE	No	PO	Northeast Agricultural University (China)	Immunogenicity and virus-neutralizing activity of swine sera *in vitro*	Zheng et al. 2021
	Bacterial vector *(Lactobacillus casei)*	S1	No	PO	Northeast Agricultural University, Jiangsu Hanswine Food Co., Biological Technology Co., Ltd (China)	Immunogenicity in mice	Xiao et al. 2022
	Bacterial vector *(L.* paracasei)	S1	No	PO	Agricultural University, Hebei Normal University of Science and Technology (China)	Immunogenicity in mice and pigs and protective efficacy in pigs	Li et al. 2024f
	Viral vector (Ad 5)	COE (genogroup GIIb)	No	IM, IN	China Agricultural University (China)	Immunogenicity in mice and virus-neutralizing activity of mice sera *in vitro*	Yan et al. 2024
	Viral vector (Ad 5)	S (genogroup GIIb)	No	IM, IN	Southwest Minzu University (China)	Immunogenicity and protective efficacy in pigs	Song et al. 2024a
	Viral vector (Ad 5)	S and S1 (genogroups GIIa and GIIb)	No	IM, PO	Lanzhou Veterinary Research Institute, Chinese Academy of Agricultural Sciences (China)	Immunogenicity in mice and virus-neutralizing activity of mice sera *in vitro*	Miao et al. 2023
	Viral vector (Pseudorabies virus)	Epitopes of S1 subunit	No	IM	Henan Agricultural University, Health Supervision Institute (China)	Immunogenicity and protective efficacy in pigs and virus-neutralizing activity of swine sera *in vitro*	Jiao et al. 2024
	mRNA	S or Sm	No	SC or IM	Institute of Veterinary Medicine and others (China)	Immunogenicity in mice and pigs, virus-neutralizing activity of mice and swine sera *in vitro* and protective efficacy in pigs	Zhao et al. 2024
TGEV+ PEDV	Chimeric VLP based on ADDomer platform	Neutralizing epitopes of TGEV (S1 subunit) and PEDV (S1 and S2 subunits)	ISA 201VG	IM	South China Agricultural University, Guangdong Academy of Agricultural Sciences (China)	Immunogenicity in pigs and virus-neutralizing activity of swine sera *in vitro*	Du et al. 2023
	circular RNA	S1 PEDV and S1 TGEV	No	IM	Shanxi Agricultural University; Shanxi Academy of Advanced Research and Innovation (China)	Safety, immunogenicity in mice and virus-neutralizing activity of mice sera *in vitro*	Zhang et al. 2025
PDCoV	Subunit	S1	Gel 01	IM	Institute of Veterinary Medicine, Jiangsu University, Agricultural University, and others (China)	Immunogenicity in mice and pigs, virus-neutralizing activity of mice and swine sera *in vitro* and protective efficacy in pigs	Chen et al. 2024
	VLP	S, M and E	No	IM	Nanyang Normal University, Northwest A&F University (China)	Immunogenicity in mice and virus-neutralizing activity of mice sera *in vitro*	Liu et al. 2023
	Subunit	RBD	Al(OH)3 or CPG2395 or aqueous adjuvant	IM	Henan Agricultural University (China)	Immunogenicity in mice	Wang et al. 2023
	Subunit	RBD dimer	Ferritin + ISA 201VG	IM	Shanghai Veterinary Research Institute (China)	Immunogenicity and protective efficacy in mice, virus-neutralizing activity of mice sera *in vitro*	Wang et al. 2024b
	VLP	S, E, M	M10	IM	Institute of Veterinary Medicine, Jiangsu University, Agricultural University, and others (China)	Immunogenicity in mice and pigs, virus-neutralizing activity of mice and swine sera *in vitro* and protective efficacy in pigs	Zhang et al. 2024b
	mRNA	S and S protein ectodomain	No	IM	Institute of Veterinary Medicine, Jiangsu University, Agricultural University, and others (China)	Immunogenicity in mice and pigs, virus-neutralizing activity of mice and swine sera *in vitro* and protective efficacy in pigs	Li et al. 2024c

IM: Intramuscular; IN: intranasal; SC: Subcutaneous; PO: by mouth (per oral), * - selection of protective epitopes and vaccine design.

This review examines coronavirus infections in livestock and poultry, which pose a major challenge to animal farming, and considers their zoonotic potential and epidemiological characteristics. There will also be a discussion of the limitations of existing vaccines and an exploration of the latest advancements and trends in veterinary vaccines aimed at preventing coronavirus infections in farm animals.

## 2. Structural features of coronaviruses: functions and antigenic properties of structural proteins

**Figure 1.**
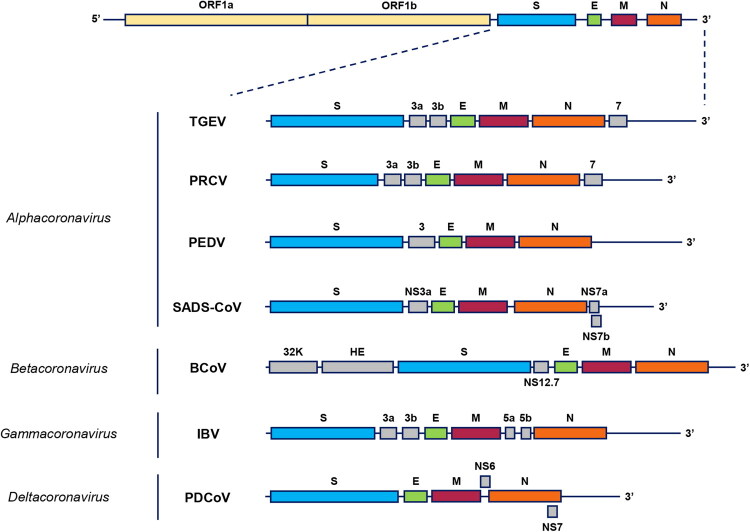
Schematic diagram showing the genomic organization of farm animal coronaviruses. Adapted by Ghosh and Malik (2020).

Coronaviruses are enveloped viruses with a spherical or pleomorphic shape, measuring 80–125 nm in diameter. A distinctive feature of CoVs is the spike-like projections on the virion surface, which resembles the spikes of a crown. Genomic (+) RNA (27–32 kb) is packed into a nucleocapsid with helical symmetry (Cui et al. 2019). Coronavirus virions contain four major structural proteins: the spike (S) protein, which forms trimers, the envelope (E) protein, the membrane (M) protein and the nucleocapsid (N) protein (Figure 1). Virions of some betacoronaviruses, particularly BCoV and PHEV, also carry an additional surface glycoprotein, hemagglutinin-esterase (HE), which forms short spikes on the virion surface. The E protein is a small hydrophobic membrane protein that plays a crucial role in virus assembly and release. The M protein is also essential for virion formation. The phosphorylated N protein is an RNA-binding protein that forms the nucleocapsid with viral RNA. The HE protein enhances S-protein-mediated cell entry. The S protein is key in viral attachment to host cell receptors and subsequent cell entry (King and Brian 1982; Westerbeck and Machamer 2019; Lang et al. 2020; Li et al. 2024a).

Receptor recognition and adsorption to the cell surface are the first step in viral infections, determining host range and tissue tropism. The S protein consists of two functional subunits: the S1 subunit mediates receptor binding and contains the receptor-binding domain (RBD), while the S2 subunit drives membrane fusion between the viral and host cell membranes. Despite their structural similarities, coronavirus S-proteins utilise a diverse array of host cell receptors and co-receptors for cell entry, and alternative receptors may be used for adaptation to new hosts and for interspecies transmission (Li 2015; Eslami et al. 2022; Zhuang et al. 2024). For example, viruses such as TGEV, PRCV, PDCoV, and possibly PEDV use aminopeptidase N as a receptor. BCoV, IBV, PHEV, and TGEV recognize different sialic acid derivatives. Specific receptors for the recently identified SADS-CoV have not yet been identified (Wickramasinghe et al. 2014; Zhuang et al. 2024).

The interaction between the S protein and host receptors is a critical factor in overcoming species barriers. The S protein serves as the primary antigen for eliciting virus-neutralising antibodies (Delmas et al. 1990; Vautherot et al. 1992; Du et al. 2009; Wickramasinghe et al. 2014; Makadiya et al. 2016; Okda et al. 2017). Consequently, it is the main component in the development of vaccines for both farm animals and humans (Kovalenko et al. 2023).

## 3. Coronavirus infections of farm animals

### 3.1. Bovine coronavirus

Bovine coronavirus is widespread globally and primarily causes intestinal and respiratory diseases in cattle, leading to significant economic losses in the livestock industry (Saif 2010; Ellis 2019; Soules et al. 2022; Berge and Vertenten 2024). BCoV was first identified in the United States, in 1972, in calves with diarrhea and subsequently appeared in Asia, Europe, across the islands of Oceania and in Africa (Stair et al. 1972; Zhu et al. 2022). After 2010, BCoV adopted an increasing epidemic trend, in many countries across five continents. The virus can be excreted from the body for an extended period – up to 932 days. This was demonstrated in calves infected with the BCoV Kumamoto/1/07 strain, indicating persistent infection (Kanno et al. 2018). Clinical manifestations of BCoV infection lead to reduced growth rates in feedlot cattle, a prolonged decline in milk production in adult cattle and high morbidity and mortality rates in calves (Saif 2010).

**Figure 2.**
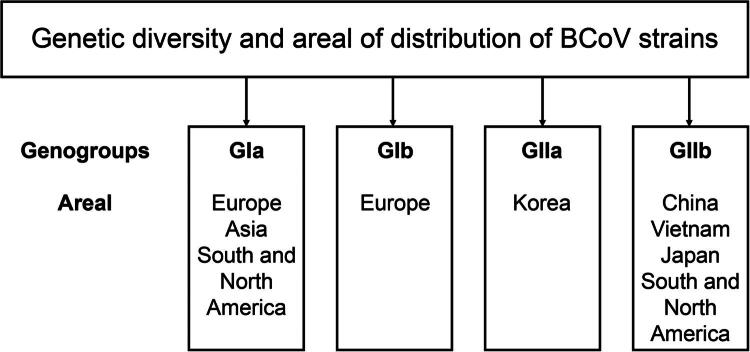
Geographical distribution of BCoV genogroups. The genotypes were classified based on a phylogenetic analysis of IBV strains using S gene sequences.

Based on clinical signs, BCoV can be divided into two groups: (1) enteric BCoV, which causes neonatal diarrhea in calves and winter dysentery in adult cattle, and (2) respiratory BCoV, which has been isolated from animals of all ages with respiratory symptoms. Studies of the geographical distribution of various BCoV isolates made it possible to initially divide them into two groups: European (GI) and Asian-American (GII). Further epidemiological studies and genomic analysis of the strains revealed their genetic diversity, with two subgroups being identified: a GIa subgroup, which included some strains from Europe, Asia and America, as well as the original Mebus strains, and a GIb subgroup, which included only European strains. Korean CoV strains were independently allocated to subgroup GIIa, while some BCoV strains from America, China, Vietnam and Japan formed the GIIb subgroup (Zhu et al. 2022) (Figure 2). Information on the circulation and genetic diversity of BCoV in the African region is currently limited. However, a study conducted in Namibia among cattle and wild ruminant populations demonstrated a relatively high prevalence of the virus and revealed the presence of a unique Namibian clade, which is of considerable interest for further study (Molini et al. 2025).

All BCoV isolates belong to the same serotype. Phylogenetic analysis of the S-protein resulted in a classification consisting of fourteen BCoV genotypes (Suzuki et al. 2020). Despite their different clinical manifestation and tissue tropism, comparative genomic and phylogenetic analyses of strains have been unable to differentiate them on the basis of a specific genetic marker into enteric or respiratory BCoV (Suzuki et al. 2020; Zhu et al. 2022; Glotov et al. 2023).

Currently, it is generally assumed that the clinical manifestations of the disease are not the result of infection with a specific strain of BCoV, but are caused by a number of other factors, such as stress, temperature, the presence of coinfections with intestinal and respiratory pathogens, and the age and health status of animals.

BCoV is transmitted by faecal-oral and aerosol routes, and potentially through contaminated surfaces, on which the virus remains infectious for 24 h (Furlan et al. 2025). Both clinically and subclinically infected animals can act as a reservoir of BCoV, and repeated infections within the herd are possible, as is transmission of the virus to another, geographically distant, herd without the need for direct contact (Liu et al. 2006).

There is various evidence that BCoV is capable of interspecies transmission, and that it causes diseases in a variety of animals. BCoV is known to affect a wide range of mammals, which are most vulnerable to this infection; more than two dozen species are affected, and these are predominantly domestic and wild ruminants (Vlasova and Saif 2021). BCoV-like strains have been detected in the faeces and in the mucous membranes of the respiratory organs of these animals. Frequent BCoV recombination events have been confirmed in several studies and they may be responsible for alterations in tissue tropism and host specificity (Bidokhti et al. 2013; Lu et al. 2017; Keha et al. 2019). Although cattle are generally considered to be the main host of BCoV, this virus has been found in camels and dogs, which can potentially act as intermediate hosts for human transmission (Corman et al. 2018). BCoV has recently been detected in China in rodents, specifically in daurian ground squirrels (*Spermophilus dauricus*) captured near cattle farms. The nucleotide identity of the full-length genome of the virus found in daurian ground squirrels in comparison with other BCoV was 97.2%–99.4% (Xu et al. 2023).

The possibility of human infection was identified in a child with acute diarrhea, in whom the coronavirus strain HECV-4408 was isolated. HECV-4408 is genetically and antigenically closer to BCoV than to the human coronavirus, HCoV-OC43. The homology of the nucleotide sequences of the S and HE genes and the amino acid sequences of the corresponding proteins between the virulent wild-type BCoV and HECV-4408 was more than 99% (Zhang et al. 1994). HECV-4408 is able to infect, and cause diarrhea in, seronegative calves, the infection of the animals resulting in 100% protection against infection with another virulent strain, BCoV-DB2 (Han et al. 2006). Based on comparative genomic and phylogenetic analyses, it was hypothesised that the human coronavirus HCoV-OC43 originated from an ancestral BCoV strain that crossed the interspecies barrier. It is assumed that this virus could have been the cause of the 1889–1890 pandemic attributed to the influenza virus (‘Russian influenza’) (Vijgen et al. 2005). There are no data to identify the causative agent in biological samples. Nevertheless, the receptor identity of HBV and OC43 (N-acetyl-9-O-acetylneuraminic acid), their serological similarity and nucleotide identity of 96% serves as additional indirect evidence supporting the possible cross-species transmission of coronavirus to humans (Vlasak et al. 1988; Kin et al. 2016). Medical reports of the clinical manifestations of disease in patients infected during the pandemic indicate a number of characteristics shared with COVID-19 (Brüssow and Brüssow 2021). In particular, they identify more severe central nervous system disorders than are typically seen during influenza outbreaks. The neurotropism of HCoV-OC43 has been shown in a number of studies (Yeh et al. 2004; Jacomy et al. 2006; Nilsson et al. 2020; Kasereka and Hawkes 2021). Recently, BCoV was detected in the brain tissue of a calf with neurological disorders (Yilmaz et al. 2024). This indicates that the risk of zoonotic transmission and the spread of BCoV to humans is high enough to pose a public health threat.

### 3.2. Infectious bronchitis virus

Infectious bronchitis virus (IBV) is the dangerous pathogen of a highly contagious disease known as avian infectious bronchitis. The disease most commonly affects chickens. IBV was discovered in the 1930s in the United States, in chickens. Since then, the disease has spread throughout the world where there are industrial poultry farms. The virus is transmitted by the aerosol route or by direct contact with contaminated surfaces. After primary infection in the respiratory tract, the virus can spread systemically and infect the kidneys and the reproductive and nervous systems, causing multiple symptoms of disease. The severity of infection depends on the specific strain of virus and the immune status of the host. Severe cases can result in high mortality rates (100%), reduced productivity and egg loss. These problems result in significant economic losses in the poultry industry (Colina et al. 2021; Zhao et al. 2023).

**Figure 3.**
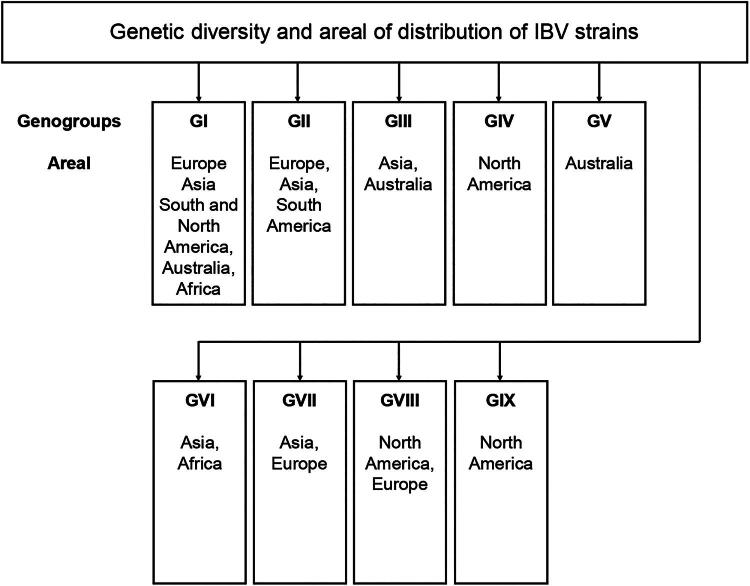
Geographical distribution of IBV genogroups. The genotypes were classified based on a phylogenetic analysis of IBV strains using S1 gene sequences (Valastro et al. 2016; Rafique et al. 2024).

IBV strains are classified into serotypes (based on cross-virus neutralising tests) and genotypes (Figure 3). A classification system based on sequence analysis of the S1 subunit has been used to determine the genotype since 2016 (Valastro et al. 2016). To date, nine genotypes (GI to GIX) and 41 genetic lines are known (Rafique et al. 2024). In most geographic regions, there is co-circulation of several IBV genotypes, of which one or two demonstrate dominance in the population, but may be periodically replaced by other genotypes or genetic lines (Jackwood and Jordan 2021; Marandino et al. 2023; Ojkic et al. 2024; Lunge et al. 2025; Salles et al. 2025; Wang et al. 2025). The most widespread genotype in the world, genotype GI, includes 31 lines. Among them, the most common genetic lines found on different continents are GI-1, GI-12, GI-13, GI-14, GI-16, GI-19 и GI-23. The distribution of other lines is regionally restricted. Genotypes GII and GIII are represented by 2 lines, while each of the other genotypes has only one line. Genotype GII has been identified in Europe and South America, genotypes GIII and GV predominate in Australia with very few reports from Asia. Genotypes IV, VIII and IX originate from North America, while GVII has been found in Europe and China (Rafique et al. 2024). A recently published study by Strydom and Abolnik (2025) identified two IBV genotypes in South Africa that had not previously been detected in the region. The first, the Brazilian strain GI-11, has not previously been detected outside of South America. The second genotype, GVI-1, was known to be limited to Asia, but its detection in South Africa expands the geographic range of the virus. Imported infected poultry is suggested to be the likely source of the emergence of GI-11 and GVI-1 strains in South Africa (Strydom and Abolnik 2025) (Figure 3).

Viruses of various GI genotype lines that are grouped with European and Chinese strains, as well as IBV variants that do not belong to any of the known genetic lines, are circulating in the Russian Federation (Bochkov et al. 2006; Scherbakova et al. 2018; Ovchinnikova et al. 2020; Marchenko et al. 2022). There is no direct correlation between genotypes/lineages and serotypes (Feng et al. 2017). More than 50 serotypes had been identified by 2000 (Ignjatović and Sapats 2000) but new variants of the virus continue to emerge due to the high frequency of mutations and recombinations. Recombination can occur among different genotypes and lines of both field and vaccine strains of IBV. Recombinant variants of IBV have been reported in various countries around the world (Ammayappan et al. 2008; Bali et al. 2021; Gong et al. 2022; Kim et al. 2023; Farooq et al. 2024; Xiong et al. 2024; Wang et al. 2024a; Lu et al. 2024b; Alhafufi et al. 2025; Strydom and Abolnik 2025).

In 2016, a new recombinant ahysx-1 isolate causing enteric disorders in chickens was discovered in China. Further studies showed a high degree of homology of this isolate with other recombinant IBV isolates collected from different provinces in China (98.0-99.8% in amino acid sequence). The 13 new recombinant IBVs were compared with 68 IBV sequences of different genotypes, as well as turkey and guinea fowl coronaviruses. The results of recombination analysis established that the main genome donors of the new isolates were IBV genotypes GI-19 or GVI-1, and donors of incorporated S protein sequences were turkey and guinea fowl coronaviruses (Wang et al. 2024a). The spread of such new recombinant viruses with pandemic potential may complicate disease prevention and control.

IBV-like coronaviruses have been isolated from a variety of poultry species, such as turkeys, pheasants and guinea fowl, with varying symptoms and disease severity. These coronaviruses are economically important in regions where commercial poultry production is developed. IBV-like viruses have also been isolated from clinically healthy representatives of wild birds, such as quails, partridges and peacocks, as well as from representatives of other orders, including wild ducks, grey geese, swans, teals and blue pigeons (Ismail et al. 2003; Cavanagh 2005; Sun et al. 2007; Felippe et al. 2010). These studies have indicated a wide range of IBV hosts among domestic and wild birds, a high probability of interspecies transmission and the possibility of long-distance spread by migratory birds. It should also be noted that specific antibodies against IBV have been detected in the blood serum of poultry farm workers, especially those who carry out the process of aerosol vaccination of chickens (Ardicli et al. 2022).

### 3.3. Porcine coronaviruses’ morbidity rates

There are currently six coronaviruses circulating in pigs, four of which cause acute gastroenteritis, with similar clinical manifestations. These include: porcine transmissible gastroenteritis virus, porcine epidemic diarrhea virus, swine acute diarrhea syndrome coronavirus and porcine deltacoronavirus. Diseases caused by porcine enteric coronaviruses (TGEV, PEDV, SADS-CoV and PDCoV) cannot be differentiated on the basis of clinical symptoms. These viruses can cause high mortality, especially in newborn piglets born to seronegative sows. Porcine respiratory coronavirus (PRCV) causes respiratory disease, while porcine hemagglutinating encephalomyelitis virus (PHEV) causes neurological, enteric and/or respiratory disorders. These viruses are transmitted *via* the fecal-oral and/or airborne routes, as well as through direct contact with contaminated surfaces. TGEV, PRCV, PHEV and PEDV have been circulating in pig populations for several decades. PDCoV and SADS-CoV are considered to be emerging CoVs, having originally been detected in pig populations in China. TGEV, PRCV, PEDV and SADS-CoV belong to the genus *Alphacoronavirus*, while PHEV and PDCoV belong to the genera *Betacoronavirus* and *Deltacoronavirus*, respectively (Table 1).

#### 3.3.1. Transmissible gastroenteritis virus and porcine respiratory coronavirus

TGEV was the first coronavirus identified in pigs, during an outbreak in the United States in the 1940s (Doyle and Hutchings 1946). This virus has spread rapidly throughout the world, affecting many countries in Asia, Europe, Africa and South America (Enjuanes and van der Zeijst 1995). Until the 1980s, TGEV infections posed a serious threat to pig production in many countries, being a common cause of enteric disease and high mortality in piglets, despite the use of vaccines. Subsequently, however, mortality rates and TGEV detection rates in pigs with diarrhea decreased significantly. One of the reasons for the decline in TGEV incidence was the emergence of its deletion mutant, known as PRCV, which is characterised by reduced virulence and tropism for the respiratory tract. This TGEV variant with a deletion in the gene encoding the S protein (621–681 nucleotides) was first reported in Belgium, in 1984 (Pensaert et al. 1986; Rasschaert et al. 1990). PRCV-induced infection is, in most cases, subclinical or mild with respiratory symptoms (Enjuanes and van der Zeijst 1995). Long-term circulation of PRCV in pig populations worldwide has resulted in a decline in the incidence of TGEV but the cross-reactivity of TGEV and PRCV antibodies significantly complicates the diagnosis and assessment of the epidemiological situation associated with TGEV. Nevertheless, new virulent TGEV strains resulting from recombination between the Purdue and Miller strains have been identified and characterised in China (Zhang et al. 2017; Guo et al. 2020). A new swine enteric coronavirus (SeCoV) has been discovered in Europe. SeCoV resulted from the recombination between TGEV and PEDV. The SeCoV S protein gene has more than 90% homology with PEDV, while the rest of the genome demonstrates up to 97% identity with virulent TGEV strains (Akimkin et al. 2016; Belsham et al. 2016; Boniotti et al. 2016; De Nova et al. 2020).

Wild and domestic animals such as foxes, dogs, mink and cats are considered as potential subclinical carriers of TGEV, acting as viral reservoirs between seasonal epidemics. However, only the virus isolated from TEGV-infected dogs has been confirmed as being infectious to pigs (Saif and Sestak 2006).

A study conducted by Vlasova et al. in 2022, reported the isolation of a new CoV variant, canine coronavirus HuPn-2018 (CCoV-HuPn-2018), in patients hospitalised with pneumonia in Malaysia. Most of the patients were children living in rural areas and frequently being in contact with domestic and wild animals. Comparative whole-genome analysis revealed a high degree of identity of the nucleotide sequences of CCoV-HuPn-2018 with several strains of canine coronavirus (90.63%–93.31%), as well as with the virulent Purdue strain of TGEV (91.47%). A phylogenetic tree based on whole-genome analysis comprised the well-supported monophyletic cluster of viruses including CCoV-HuPn-2018, other canine and feline coronaviruses and porcine coronaviruses TGEV and SeCoV, proving the close genetic relationships between these viruses (Vlasova et al. 2022). The results presented in the study not only reported the identification of a novel recombinant CoV in humans, which arose from multiple recombination events between different *Alphacoronavirus 1* strains, with a recent zoonotic origin, but also acted as a worrying harbinger of possible future public health challenges.

#### 3.3.2. Swine acute diarrhea syndrome coronavirus

Swine acute diarrhea syndrome coronavirus (SADS-CoV) was originally detected in 2017, in several pig farms in Guangdong Province, China. This virus caused a large outbreak that killed approximately 25,000 animals (Gong et al. 2017; Pan et al. 2017; Zhou et al. 2018). Clinical manifestations were similar to those caused by other enteric CoVs in pigs, and included severe diarrhea and acute vomiting, leading to death due to rapid weight loss in neonatal piglets. Mortality rates reached 90% among piglets under five days of age (Zhou et al. 2018). In subsequent years, in 2018, 2021 and 2023, repeated outbreaks associated with SADS-CoV were reported on farms in China (Zhou et al. 2019; Sun et al. 2022; Zhang et al. 2024a). Comparative genomic analysis of isolates obtained in different years showed ∼99.9% identity with the first SADS-CoV isolate, obtained in 2017 (Hassanin et al. 2024; Zhang et al. 2024a). Phylogenetic analysis allowed suggesting that all SADS-CoV strains have a common origin from a single source: *Rhinolophus affinis* bats living in Guangdong Province. This virus has been spreading among pig farms, in several provinces of China, for seven years after the first outbreak (Zhou et al. 2018; Hassanin et al. 2024). To investigate the potential transmission of SADS-CoV from animals to humans, serum samples from farm workers who had close contact with infected pigs were tested for antibodies to the virus. No SADS-CoV-specific antibodies were detected in any of the samples (Zhou et al. 2018). Studies by Edwards et al. (2020), however, revealed the zoonotic potential of SADS-CoV for transmission to humans. Despite that fact that SADS-CoV did not use human coronavirus ACE-2, DPP4, or CD13 receptors for docking and entry, researches showed that SADS-CoV replicated efficiently in primary human respiratory and intestinal cells, indicating human susceptibility to infection and the potential for animal-to-human transmission (Edwards et al. 2020).

#### 3.3.3. Porcine hemagglutinating encephalomyelitis virus (PHEV)

The disease known as porcine hemagglutinating encephalomyelitis was first reported in 1957, in Canada, in piglets presenting a vomiting syndrome followed by neurological damage (Roe and Alexander 1958). However, the virus causing this disease was not isolated and characterised until 1962, during an outbreak of encephalomyelitis in newborn piglets (Greig et al. 1962). PHEV is the only known neurotropic coronavirus that infects pigs. Later, PHEV was detected in many countries, including Belgium, the Czech Republic, the United States, Argentina, South Korea and China (Mora-Díaz et al. 2019; Moutelikova and Prodelalova 2019). In piglets over four weeks of age and in adults, the infection is usually subclinical. For newborn piglets, the mortality rate can reach 100%, depending on the presence of specific antibodies in the sow’s colostrum (Mora-Díaz et al. 2019). However, PHEV has a relatively minor impact on pig populations, compared with other pathogenic coronaviruses.

For a long time, there was little information, in scientific literature, about the epidemiology and genetic diversity of strains of this virus. Recent epidemiological studies conducted in the United States, South Korea and China have provided strong evidence that PHEV is widespread in pig populations and is characterised by the high genetic diversity of strains (Mora-Díaz et al. 2020; Kim et al. 2022; Shi et al. 2024). In addition, new PHEV variants have been identified that can cause respiratory disease in young and adult pigs (Lorbach et al. 2017; He et al. 2023).

#### 3.3.4. Porcine deltacoronavirus

Porcine deltacoronavirus, or PDCoV, was first identified in 2012, as a result of molecular genetic monitoring of avian and mammalian coronaviruses in Hong Kong (Woo et al. 2012). Its pathogenic effect on pigs was, however, unknown until 2014. In 2014, PDCoV was identified as an etiological agent of diarrhea outbreaks, which began in the U.S. state of Ohio and quickly spread to 20 other states (Wang et al. 2014; 2016a). In subsequent years, PDCoV was recorded in many countries, such as Canada, Peru, Mexico, Japan, mainland China, Thailand, Vietnam, Laos and South Korea, causing significant economic damage in the pig farming sector (Suzuki et al. 2018; He et al. 2020). The symptoms caused by PDCoV are similar to those of PEDV and TGEV. These include vomiting, dehydration and diarrhea. The mortality rate among infected piglets is approximately 30%–40%. However, PDCoV co-infection with other swine coronaviruses - PEDV, TGEV and/or rotavirus – can lead to more serious consequences (Liu and Wang 2021).

Epidemiological studies based on the analysis of full-length PDCoV genomes (119 isolates) collected over a period of fifteen years, worldwide, have identified four PDCoV phylogenetic lineages, with different geographical distributions. The ‘Thailand’ lineage includes strains found in Vietnam, Laos and Thailand. The lineages ‘Early China’ and ‘China’ are represented by strains circulating exclusively on the territory of China, while the ‘USA’ lineage unites strains that were identified in the USA and subsequently spread to Japan, Korea and China (He et al. 2020). Further studies and strains genomic analysis (166 isolates) have enabled the distinction of two genogroups, GI and GII, which have been divided into seven (GIa-g) and two (GIIa, GIIb) subgroups, respectively (Bahoussi et al. 2022). Chinese isolates are represented in all subgroups and have demonstrated more frequent recombination and higher genetic diversity than isolates from other regions (He et al. 2020; Bahoussi et al. 2022).

There is evidence that PDCoV is capable of interspecies transmission and can cause diseases in various animals. In particular, experimental PDCoV infection of mice (Zhang et al. 2022a), calves (Jung et al. 2017) and poultry (chickens and turkeys), followed by transmission to healthy individuals, has been recorded (Liang et al. 2019; Boley et al. 2020). PDCoV has been identified in the faeces of wild birds, Asian leopard cats and badgers (Woo et al. 2012). In addition, PDCoV has been found to be able to infect various cell lines derived from bulls, chickens, dogs and humans, including human intestinal epithelial cells (Cruz-Pulido et al. 2021; Li et al. 2024c). Interspecies transmission of PDCoV to humans was recorded, recently, in rural areas of the Republic of Haiti, an island nation in the northern Caribbean. In a study conducted by Lednicky et al. in 2021, three PDCoV strains were detected in blood plasma samples from children suffering from acute undifferentiated febrile illness, at different times between 2014 and 2015. It is assumed that the zoonotic potential of PDCoV in the human population, especially in rural areas where contact with domestic animals is common, is very high (Lednicky et al. 2021).

#### 3.3.5. Porcine epidemic diarrhea virus

Porcine epidemic diarrhea outbreaks were first reported on British farms in 1971, and the etiological agent of the CV777 disease was identified, in 1978, in Belgium as a new coronavirus (Pensaert and de Bouck 1978), which is currently considered a classic PEDV strain. In the 1970-1980s, outbreaks in various European countries caused serious economic damage. From the 1980s to 2010s, the spread of PEDV was mainly limited to Europe and Asia, causing only sporadic outbreaks (Song and Park 2012). A highly virulent pandemic variant of PEDV, which differed from the classic CV777 strain, appeared in China, in 2010, for the first time (Li et al. 2012). A major outbreak quickly spread across the country, causing devastating damage to pig farming. The mortality rate reached 100% in newborn piglets, even on farms where vaccination was carried out. The inactivated vaccine based on the CV777 strain proved ineffective. In 2013, the same variant of PEDV caused a pandemic in the USA, reducing the animal population by 3% and causing enormous losses (Weng et al. 2016). By the end of 2016, new strains of PEDV had spread across North America, Asia and Europe. Today, PEDV continues to be a serious threat to the pig industry in most countries and regions of the world.

**Figure 4.**
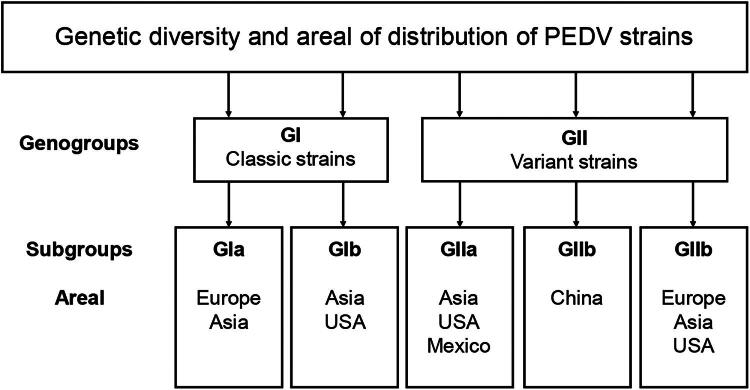
Geographical distribution of PEDV genogroups. The genotypes were classified based on a phylogenetic analysis of PEDV strains using full-size genome sequences.

Based on full-length genomes, PEDV strains were classified into two genogroups: GI (classical strains) and GII (variant strains). These genogroups include two and three subgroups, respectively: GIa-b, GIIa-c (Wang et al. 2016b; Guo et al. 2019; Zhang et al. 2022a) (Figure 4).

Early European strains (virulent CV777 and DR13) and Chinese classic strains belong to the GIa subgroup, whereas the GIb subgroup mainly consists of cell culture-adapted vaccine strains (attenuated CV777 and DR13) and other pandemic classic strains (AH-M, SD-M, SQ2014 and SC1402). Unlike Chinese strains of the GIb subgroup, strains from the USA are characterised by the presence of small insertions and deletions in the S gene, which are also known as S-Indel strains (Vlasova et al. 2014).

Genogroup GII includes variant strains that appeared in 2010, which are now prevalent worldwide, especially in China. The GIIa subgroup includes strains with higher virulence than the GI genogroup, which are common in Asia and North America. All virulent strains of the GIIb subgroup are currently circulating in China (Zhang et al. 2022b). The GIIc subgroup appeared as a result of recombination events between strains of the GIa (S-Indel) and GIIa subgroups. Less virulent strains of the GIIc subgroup are mainly distributed in Europe, the United States and China (Guo et al. 2019; Peng et al. 2024). By the end of 2016, PEDV variant strains had spread all over North America, Asia and Europe. In addition, recombinant SeCoV (see 3.3.1), which appeared as a result of recombination between TGEV and PEDV, were detected in Europe at about the same time. Today, PEDV continues to be a serious threat to the pig industry in most countries and regions of the world (Lei et al. 2024; Makau et al. 2024).

Data indicating that PEDV is capable of interspecies transmission are limited. PEDV has been detected only in wild pigs in Korea and the United States, with transmission occurring from domestic animals (Lee et al. 2016; Bevins et al. 2018). However, PEDV is able to infect cell cultures derived from humans, monkeys and bats, which confirms the hypothesis that the virus once crossed the interspecies barrier and passed from bats to pigs (Liu et al. 2015).

## 4. Vaccines against economically significant coronavirus infections in farm animals

### 4.1. Vaccines against bovine coronavirus infection

Live attenuated and inactivated vaccines aimed at preventing neonatal diarrhea in calves have been used for half a century. Maternal antibodies partially protect calves from diarrhea in the first months of life, when the virus is most dangerous. Although monovalent vaccines against BCoV exist, combination vaccines are now widely used. One of these vaccines is Calf-Guard^®^ (Zoetis Inc.), which contains attenuated strains of coronaviruses and rotaviruses. This vaccine can be effective when administered orally to newborn calves in the first hours of life. Another vaccine, Scour-Guard^®^ 4KC (Zoetis Inc.), represents the same viral mixture complemented with *Clostridium perfringens* type C beta-toxoid and inactivated cells of the enterotoxigenic *E. coli* strain. The latter vaccine is intended for parenteral administration to in-calf cows to induce passive lactogenic immunity. Inactivated combined vaccines preventing the spread of coronavirus-caused infectious diseases in cattle are used in the Russian Federation (Table 1). Among these, the FGBI «ARRIAH»-developed preparation is administered to prevent rotavirus and coronavirus infections in animals. Other Russian inactivated multicomponent combined vaccines include the Kombovak series (Kombovak, Kombovak-A, Kombovak-K), of various compositions, produced by Vetbiochem LLC. These preparations are designed to protect cattle from intestinal and respiratory infections caused by both viruses and bacteria.

Vaccination programmes, despite being widely implemented in many countries, cannot completely prevent viral particles from spreading into the environment after infection. Both adults and young animals, while being carriers, are known to pass on the virus, even while showing no symptoms of the disease, which presents significant challenges for infection control (Hodnik et al. 2020). The effectiveness of commercial vaccines against respiratory form of neonatal diarrhea is very poorly covered in the literature and the data presented are contradictory. The intranasal administration of a BCoV/rotavirus combined live attenuated vaccine has been shown to reduce the risk of respiratory disease in calves released to the feedlot (Plummer et al. 2004). However, a recent study of the prime boost intranasal vaccination followed by intramuscular vaccination using the same combined BCoV/rotavirus vaccine showed no significant difference in efficacy compared to a control group. At the same time, intranasal prime-boost immunisation of calves with a monovalent commercial live attenuated vaccine (Merck Animal Health, Kirkland, Quebec) has been found to significantly increase the efficacy of preventing respiratory coronavirus infection (Erickson et al. 2024). These results, together with those relating to the efficacy of the combined vaccine against other cattle respiratory diseases induced by herpesviruses, such as bovine herpes virus type 1, bovine respiratory syncytial virus and others, suggest encouragement for further research (Erickson et al. 2024).

Commercial vaccines may also lack effectiveness due to the genetic and antigenic discrepancy between vaccine strains and those currently in circulation. In particular, South Korea has a high incidence of neonatal calf diarrhea and adult winter dysentery, which has been found to be caused by common BCoV strains that belong to the GIIa subgroup, yet vaccinations have been carried out using a strain from the GI group. Recently, a live attenuated vaccine has been developed in Korea, based on a Korean strain from the GIIa subgroup, and preliminary results have been obtained in relation to the viral tropism in the gastrointestinal tract of calves after oral administration. The Korean vaccine strain has 13 amino acid substitutions in the S-protein and has been tested for reversion to a virulent strain. The authors plan to conduct research on the protective properties of the candidate vaccine (Park et al. 2024).

A number of research teams have focused on creating recombinant BCoV vaccines using various approaches emerging from the development and licensing of vaccines during the COVID-19 pandemic. These include the bioinformatics-based selection of protective epitopes and the rational design of polyepitope vaccines (Table 2) (Awadelkareem and Hamdoun 2024; Duraisamy et al. 2024; Jiang et al. 2024; Pratelli et al. 2024; Rani et al. 2024; Yu et al. 2024a). It is worth mentioning that this approach to creating a recombinant BCoV vaccine emerged only after the pandemic.

There is a trend for developing an adenovirus vector vaccine encoding the genes of the BCoV M and S structural proteins (Pratelli et al. 2024). A comparative analysis of three candidate vaccines, based on either one antigen (M or S) or two antigens (M + S), revealed that a vaccine containing two antigens at the same time enhances immunogenicity in sheep (Pratelli et al. 2024). Promising results have been achieved in the development of a candidate vaccine based on virus-like particles obtained by the co-expression of five BCoV structural proteins (E, M, N, S, HE) in the baculovirus expression system in insect cells. The immunisation of mice and cows with such VLPs combined with two adjuvants (MF59, CPG55.2) has resulted in the production of high titres of specific IgG in animal sera. The titres of virus-neutralising antibodies in the immunised animals’ sera were significantly higher than those obtained with the inactivated BCoV vaccine (Yu et al. 2024a). In the study by Chineka et al. (2025), amino acid sequences of BCoV strains of different genotypes obtained from different geographical regions at different times were analyzed. Based on these data, a poxvirus-based vector vaccine was developed that included consensus sequences of the structural proteins S and N. Immunization of mice with the candidate vaccine induced high levels of specific IgG antibodies to the S protein and a T cell immune response to the N protein (Chineka et al. 2025). The application of the consensus approach in vaccine development is a promising direction for the generation of universal vaccines that can induce broad-spectrum immune response. The latter is crucial to combat genetically variable viruses such as CoV. Additional studies are required to evaluate the effectiveness of the developed vaccine and assess its cross-protection. This information will facilitate further improvement of vaccine strategies against BCoV and other viruses with high genetic variability.

### 4.2. Vaccines against avian infectious bronchitis

The first vaccine against the IBV was developed in the 1950s. Currently, vaccination remains the main means of preventing and controlling IBV in poultry farming. Commercial vaccines used widely worldwide are mainly live attenuated vaccines (Table 1). Inactivated vaccines are often used as a booster to enhance the immune response. The most effective vaccination strategy is to base vaccines on viruses circulating within the population. However, the distribution of various IBV genotypes/serotypes strains in poultry farms, together with recombinant and variant isolates that do not belong to any of the known genotypes, significantly reduces the effectiveness of existing vaccines (Zhao et al. 2023). Homologous live vaccines are not available for many IBV variants. Currently, the world’s most common vaccines are live attenuated vaccines based on Massachusetts type strains (genotype GI-1), which have recently been frequently used in combination with the IBV variants most common in the region of use. In the Russian Federation, live attenuated and inactivated vaccines are used that are based on Massachusetts H-120 and H-52 strains (Avivak IBK, Avivak IBK+NB), as well as a newly registered live attenuated vaccine based on the VNIIZH 793/B strain (genotype GI-13). Thus, heterologous immunisation schemes using various vaccine strains, such as Massachusetts and 793B, have become available. The 793B genotype/serotype, discovered in the UK in the 1960s, is widespread worldwide. The combined immunisation of animals with vaccine strains such as Massachusetts and 793B has been shown to be effective in protecting against some other field IBV strains, providing a wide range of protection (Cook et al. 1999; Awad et al. 2015; De Wit et al. 2015; Belkasmi et al. 2017; De Wit et al. 2017). Vaccination with the Massachusetts type H120 strain is often used in combination with local strains such as QX (GI-19) and type 4/91 (GI-13) in China, or the endemic strain LDT3-A (GI-28) (Zhao et al. 2023). A heterologous vaccination with two vaccines, GA08 (GI-27) and Mass (GI-1), is a recommended strategy to protect against the DMV1639 (GI-27) virus, which is currently dominant in the United States and Canada (Brimer et al. 2025).

Due to the high variability of the IBV and the limitations associated with cross-protection, however, modern vaccines are unable to provide comprehensive protection against the various strains of IBV currently in circulation. It has been shown that the immunisation of chickens, even with a combination of three and four vaccine-attenuated strains, leads to only partial protection (Jackwood et al. 2020). In addition, the widespread use of live attenuated vaccines may provoke the emergence of new virulent IBV variants, thus complicating diagnostics. For example, a new virulent strain has been identified in China, resulting from the recombination of three vaccine-attenuated strains. This strain causes respiratory and kidney diseases, which lead to death in chickens (Gong et al. 2022).

However, the process of developing live attenuated vaccines corresponding to circulating strains is actively ongoing. The design of new vaccines based on virulent strains is a complex task that requires careful balancing between preserving critical antigenic determinants and ensuring maximum safety of the final product. In a study conducted by Huang et al. (2025), two recombinant vaccines (H120-FQH QX3 and H120-CSL) based on the H120 strain were obtained using reverse genetics methods, replacing the genes encoding the S1 and N proteins with the genes of the FQH strain QX3 (QX-like IBV) and the CSL strain. The FQH QX3 and CSL strains were selected based on the results of cross-neutralization tests from 6 epidemic strains circulating in China that cause outbreaks of the disease in vaccinated birds. It was shown that the antiserum to the FQH QX3 strain had the widest spectrum of neutralization, while the antibodies produced by existing vaccine strains weakly neutralized the CSL strain. The protective effectiveness of the developed vaccines after infection of birds with CSL and CY strains was evaluated based on clinical signs, histopathological examination data, parameters of ciliostasis in the trachea, dynamics of viral load in tissues, as well as a decrease in virus release from the trachea and cloaca of chickens. A commercial combined live attenuated vaccine against infectious bronchitis (strain H120) and Newcastle disease (strain LaSota) was used for comparative analysis. The H120-FQH QX3 vaccine demonstrated the lowest effectiveness in protecting chickens after infection with the CY strain (60%), despite the fact that the CY and FQH QX3 strains belong to the same serotype. The most effective vaccine in the study was the H120-CSL vaccine, which makes it a promising candidate for further research. The S1 and N antigens of the CSL strain have demonstrated high immunogenicity and, according to the authors, can become the basis for the development of new recombinant vaccines with improved characteristics (Huang et al. 2025).

The continuous emergence of new IBV variants highlights the need for innovative approaches in vaccine development. Currently, a number of research teams are actively working on the development of recombinant IBV vaccines, applying a variety of strategies to develop highly effective and safe vaccine preparations that provide a wide range of protection (Shirvani and Samal 2020; Zuo et al. 2021; Sepotokele et al. 2023a, 2023b; Liu et al. 2024).

A study of the S-protein sequences of 257 IBV strains isolated in China has created the opportunity to develop a DNA vaccine containing the consensus sequence of the S-protein ectodomain (Zuo et al. 2021). The candidate DNA vaccine induced both humoral and cellular immune responses and protected chickens from infection with the heterologous M41 strain as effectively as the homologous live attenuated H120 vaccine (China). The consensus approach is used to create vaccines against genetically highly variable viruses and enables the creation of a synthetic vaccine antigen, the amino acid sequence of which is as close to the sequences of isolates currently circulating as possible (Kondakova et al. 2021).

Another recombinant vaccine has been developed based on the *Salmonella typhimurium* strain containing a plasmid encoding the S1 subunit of the S protein (Liu et al. 2024). A strain of *S. typhimurium* χ11246 with a regulated system of delayed attenuation and lysis was used to increase the safety and effectiveness of the vaccine candidate. Oral immunisation with a recombinant strain of *S. typhimurium* induced both a humoral and cellular immune response and partially protected against infection with a homologous strain. Further research involves studying the safety of the recombinant S. strain *typhimurium* (Liu et al. 2024).

Chimeric VLPs obtained in *Nicotiana benthamiana* plants by transient expression have been used to develop a candidate vaccine in South Africa (Sepotokele et al. 2023b; 2023b). The VLPs consist of the M proteins of the Newcastle disease virus and the full-length S protein of the IBV with a number of modifications providing stability and successful assembly in plants. The VLP yield was ∼17 mg/kg of leaf material (Sepotokele et al. 2023a). The candidate vaccine was tested on target animals, demonstrating high immunogenicity and the absence of side effects. The vaccinated chickens showed reduced virus release from the oral cavity and cloaca after infection with a homologous virus, compared with the unvaccinated control group. In addition, the chickens were better protected from tracheal ciliostasis than birds vaccinated with live vaccine (Sepotokele et al. 2023a, 2023b).

### 4.3. Vaccines against porcine coronavirus infections

Live attenuated and inactivated vaccines to prevent disease caused by TGEV and PEDV are currently licensed and commercially available in many countries (Table 1). There are, however, no licensed vaccines against PHEV, PDCoV and SADS-CoV. Despite the global implementation of vaccination programmes, TGEV and PEDV are widespread in many countries around the world. Of particular concern is PEDV, since the emergence of new variants of this virus reduces the effectiveness of vaccines and requires the constant updating of strains (Kim et al. 2023). A recently published study by Lu et al. (2024a) presented results demonstrating the effectiveness of an attenuated vaccine based on the 17GXCZ-1ORF3d (GIIb) strain characterized by a truncated ORF3 gene (Lu et al. 2024a). ORF3 located between the genes of the structural proteins S and E protein (Figure 1), encodes the only accessory protein of PEDV, which is involved in viral pathogenesis, in particular, it affects the virulence and modulates the host immune response (Si et al. 2023; Zheng et al. 2023; Lu et al. 2024a). Immunization of sows with the 17GXCZ-1ORF3d-P120 vaccine demonstrated a significant increase in the levels of specific IgG and IgA and provided partial passive cross-protection of piglets from infection with heterologous strains (GIIa). These results confirm the potential of attenuated strains with truncated ORF3 genes to devise effective vaccines. With the discovery of PEDV variants resulting from recombination between the vaccine strain and the field strain, recombination-induced reversion to a virulent strain raises serious concerns regarding the use of live attenuated vaccines. To overcome this problem and develop new safe, immunogenic and genetically stable live attenuated vaccines against PEDV, researchers are turning to reverse genetics technologies (Yu et al. 2024b). In order to improve the effectiveness of inactivated vaccines against TGEV and PEDV, studies are being conducted to find the most optimal methods of drug administration and to develop new adjuvants. In particular, in China, in addition to traditional methods of administering the vaccine, the Houhai acupuncture point is used for injections (Kong et al. 2023). A study conducted by Su et al. (2023) demonstrated the efficacy of a novel mucosal adjuvant consisting of saponin derived from ginseng stems and leaves in combination with an inactivated vaccine. As is known, passive immunity acquired by piglets through the colostrum and milk of sows is the most effective method of protection, in the first days of life, from intestinal CoV. However, inactivated vaccines, despite their safety, provide only very limited lactogenic immunity. Oral administration of a mucosal adjuvant prior to parenteral vaccination with an inactivated PEDV vaccine is a promising method for inducing lactogenic immunity in sows. This approach results in a significant increase in the number of PEDV-specific IgA-producing plasma cells in the intestine and, as a result, an increase in IgA secretion into milk (Su et al. 2023). Studies are currently underway to develop experimental recombinant vaccines against PEDV and various immunisation regimens. Among these, subunit vaccines, VLP-based vaccines, vector vaccines and mRNA vaccines can be distinguished.

Live bacterial vectors represent a promising platform for expression of a target gene in the host organism upon oral administration. Recombinant strains of Lactobacillus spp. with a plasmid encoding the target antigen have been the subject of a number of studies (Zheng et al. 2021; Xiao et al. 2022). The oral administration of a recombinant *L. johnsonii* strain expressing the COE antigen (CO-26K equivalent, S protein fragment, aa 499–638) to sows resulted in the synthesis of both specific serum antibodies (IgG, IgA and IgM) and secretory immunoglobulin A (SIgA) antibodies. High levels of specific SIgA and IgG antibodies were also detected in maternal milk, which provided effective protection to piglets against infection with the virulent strain (Zheng et al. 2021). In a study conducted by Li et al. (2024b) to enhance the genetic stability of the *L. paracasei* bacterial vector, the gene encoding the target antigen (S1 subunit) was integrated into the bacterial chromosome. Oral administration of the recombinant strain to mice and piglets induced mucosal, humoral and cellular immune responses and partially protected piglets from PEDV infection (Li et al. 2024b).

The oral route of vaccine administration has also been investigated for viral vector vaccines. In a study by Miao et al. (2023), four recombinant adenoviruses of serotype 5 (Ad5) were constructed expressing the S or S1 genes of strains of two genogroups (GIIa and GIIb). A comparative assessment of their immunogenicity was then carried out upon oral and intramuscular administration in a mouse model. Mice immunised with candidate vaccines by intramuscular injection demonstrated strong humoral and cellular immune responses, with neutralising antibody titres comparable with levels achieved with an inactivated vaccine. However, oral administration to mice *via* gavage was not effective in stimulating either mucosal or systemic immunity (Miao et al. 2023). Although the fecal-oral route is generally considered to be the main route of transmission for PEDV, the potential for airborne transmission *via* disruption of the respiratory endothelial barrier has recently been identified. PEDV exhibits tropism for nasal epithelial cells and can cause severe diarrhea in suckling piglets when administered intranasally (Li et al. 2022). Song et al. (2024a) generated recombinant Ad5 expressing the S protein of a GIIb genogroup strain and compared two routes of drug administration in pregnant sows. Specific IgG and SIgA were detected in the colostrum of immunised animals, with antibody titres being higher in the group with intramuscular administration of the drug. In addition, cross-neutralising activity of serum antibodies against strains belonging to genogroups GIb and GIIa was demonstrated. The efficacy of protection of five day-old piglets against infection with a heterologous strain of the GIIa subgroup was 100% with parenteral administration of the candidate vaccine and 80% with intranasal administration (Song et al. 2024a). In a study by Yan et al. (2024), however, intranasal delivery of a recombinant vector expressing the COE antigen was found to be more effective in stimulating serum and secretory IgA synthesis in mice than an intramuscular injection (Yan et al. 2024).

Zhao et al. (2024) have developed two mRNA vaccines encapsulated in lipid nanoparticles (LNPs). The antigens chosen were S protein (GIIb subgroup strain) or Sm, a polyepitopic protein containing the N-terminal domain (aa 19–233), COE (aa 499–638) and several linear neutralising epitopes (aa 744–774) of the S protein. Mice immunised with the full-length S protein-based vaccine (S mRNA-LNP vaccine) induced higher levels of IgG and neutralising antibodies against different PEDV subtypes (GIIb, GIIa and GI) than a group of mice immunised with a Sm mRNA-LNP vaccine. Piglets immunised with PEDV-S mRNA, as well as piglets born from vaccinated sows and subsequently challenged with a homologous strain of the GIIb subtype, presented a significant reduction in the clinical signs of disease and intestinal pathological lesions. However, although vaccination significantly reduced the viral load in intestinal tissue and faeces, it was unable to completely prevent the spread of the virus (Zhao et al. 2024).

Several research groups are developing subunit vaccines based on recombinant S protein and its fragments, as well as vaccines based on VLPs obtained in various expression systems. Masuda et al. (2021) optimised the PEDV S protein sequence to produce a stable trimer in a baculovirus expression system in insect cells (silkworm-BEVS) by introducing exogenous trimerisation motifs. The yield of recombinant protein was approximately 2 mg per 10 ml of larval serum. Immunisation of mice in the presence of Montanide IMS 1313 adjuvant induced high levels of IgG and neutralising antibodies (Masuda et al. 2021). Guo et al. (2024) produced recombinant S1, COE antigens and a trimer of full-length S protein (GIIb subgroup strain) in a eukaryotic expression system in HEK-293F cells. The immunogenicity of the proteins obtained was assessed in the presence of two adjuvants. Comparative analysis showed that the combination of S protein trimer with M103 adjuvant induced the most powerful cellular and humoral immunity and the highest titre of neutralising antibodies. In cell culture virus neutralisation tests, positive results were obtained not only with the homologous strain of the GIIb subgroup, but also with the heterologous strains GIIa and S-indel. Immunisation of three day-old piglets with the S/M103 candidate vaccine also induced high levels of S-specific IgG, IgA and neutralising antibodies. Results of the homologous virus challenge test showed a significant reduction in clinical symptoms and viral load in faeces (Guo et al. 2024). A study by Song et al. (2024b) demonstrated low levels of cross-protection between GIIa and GIIb subtype strains when piglets were infected. In this regard, a bivalent subunit vaccine was developed based on trimers of full-length S proteins of two strains belonging to these subtypes. The candidate vaccine induced significant levels of neutralising IgA and IgG antibodies in sows, providing lactogenic immunity in newborn piglets and protection against challenge with GIIa and GIIb subtype strains (Song et al. 2024b).

Liu et al. (2024) conducted a comparative study of the immunogenicity and protective efficacy of an inactivated vaccine and a subunit vaccine based on PEDV S-protein expressed in mammalian cells. The results showed significantly higher titers of PEDV specific IgG and IgA and neutralizing antibodies in the blood serum of animals immunized with the subunit vaccine. To assess the protective effectiveness, piglets born from vaccinated sows were infected with a virulent homologous PEDV strain. The analysis showed that in the group vaccinated with the subunit vaccine, there was a statistically significant decrease in viral excretion, less pronounced clinical symptoms and minimal pathomorphological changes in the intestine compared to the control group receiving the inactivated vaccine (Liu et al. 2024). Recently, a group of Chinese researchers identified a new variant of PEDV (GIIb) designated as ShXXY2-2023. This variant is characterized by specific mutations in key neutralizing epitopes, including the N-terminal domain, RBD, and COE, which significantly distinguishes it from existing vaccine strains. The inactivated vaccine based on ShXXY2-2023 induced higher titers of neutralizing antibodies in sows compared to commercially available vaccines and more effective passive protection of newborn piglets from infection with heterologous strains. It has been shown that the titers of neutralizing antibodies in the blood serum of sows correlate with the survival rate of piglets after infection. Neutralizing antibodies titers from 1:377 to 1:774 or higher a week before farrowing provided piglets with protection efficiency of more than 80%. (Hu et al. 2025).

Hsu et al. (2020) developed a vaccine based on VLPs obtained by co-expression of structural proteins E, M, S (GIIb subgroup strain) in a baculovirus expression system in Sf21 insect cells. Immunisation of six month-old piglets with PEDV VLPs using mucosal adjuvants CCL25 and CCL28 together with Freund’s adjuvant, instead of immunisation without mucosal adjuvants, contributed to the induction of serum IgA synthesis and increased the production of specific IgG. In all groups of animals, immunisation was carried out in the presence of Freund’s adjuvant; without adjuvant, the immunogenicity of VLPs was not assessed. After infection with the virus, the immunised group of animals demonstrated a milder clinical course of the disease, with reduced spreading of the virus in faeces than the control group (Hsu et al. 2020). A study by Kim et al. (2021) attempted to optimise the yield of VLPs produced in a mammalian expression system. As a result, it was found that the yield of VLPs decreased in the presence of structural E protein and increased in the presence of N protein. The efficacy of VLPs produced by co-expression of the structural proteins S, M and N in mammalian HEK293T cells was compared with that of the inactivated vaccine. Although serum IgG titres were significantly higher in mice receiving the inactivated virus vaccine, IgA and virus-neutralising antibody titres were significantly higher in mice vaccinated with VLPs in the presence of an aluminum hydroxide adjuvant. In the absence of an adjuvant, VLPs’ immunogenicity was low (Kim et al. 2021). Among the new developments based on VLPs is a chimeric vaccine obtained on the ADDomer platform – multimeric proteins of the adenovirus. Each ADDomer virus-like particle is formed by the assembly of 12 pentameric protein complexes and has up to 360 potential sites for the insertion of antigenic epitopes. Short neutralising epitopes of TGEV (S1 subunit) and PEDV (S1 and S2 subunits) were selected as antigens. The immunisation of four week-old piglets with the candidate bivalent vaccine in combination with the adjuvant ISA 201VG demonstrated immunogenicity and effective stimulation of the synthesis of neutralising antibodies against both PEDV and TGEV. However, neutralising antibody titres were lower than in the control group immunised with a live attenuated vaccine (Du et al. 2023).

With the emergence and spread of novel CoVs with zoonotic potential, such as PDCoV and SADS-CoV, the development of vaccines against these viruses is of critical importance. Inactivated vaccines against PDCoV have been developed in China. These vaccines are effective in passively protecting piglets born from vaccinated sows, but they do not provide 100% protection against virulent virus challenge (Zhang et al. 2020). Various adjuvants, including aluminum hydroxide and CpG ODN (Zhao et al. 2022), and different vaccination approaches, such as vaccination of piglets born from seronegative sows (Fan et al. 2024), are being investigated to enhance the immune response and efficacy of inactivated vaccines.

Currently, the main efforts of research groups focus on the development of PDCoV vaccines. Wang et al. (2024) designed RBD-Fe nanoparticles by covalently coupling of RBD-dimer and *Helicobacter pylori* ferritin using the SpyTag/SpyCatcher system. Both RBD-dimer and ferritin were expressed in *E. coli*, RBD sequence was corresponded to the new virulent PDCoV strain (JS2211). RBD-Fe nanoparticles in combination with ISA 201VG adjuvant were shown to induce humoral and cellular immune responses in mice after two immunisations. In particular, a high level of PDCoV RBD-specific IgG and neutralising antibodies were detected. Histopathological studies and RT-qPCR for virus detection revealed a considerable reduction in the JS2211 strain virus infection rate in mice immunised with the vaccine candidate, in comparison with a control group (Wang et al. 2024b).

Chen et al. (2024) developed a subunit vaccine based on S1-subunit of PDCoV S-protein. A vaccine antigen was produced in a Bac-to-Bac Baculovirus Expression System in Ti5 insect cells. Comparative analysis of immune sera of mice immunised with two different types of vaccines - the subunit vaccine or an inactivated vaccine - resulted in comparable levels of IgG and neutralising antibodies. Similar results were observed after immunisation of gestating sows, while titres of IgA in blood and milk sera were higher for the group immunised with the subunit vaccine candidate. Piglets born from vaccinated sows were protected from CZ2020 strain challenge, as demonstrated by the absence of any clinical signs of disease, although low levels of virus RNA were detected in faeces (Chen et al. 2024).

Zhang et al. (2024b) designed a VLP-vaccine candidate against PDCoV. This VLP-vaccine candidate was based on structural protein S, M and E (CZ2020 strain) and expressed in HEK 293 T mammalian cells. An experiment was conducted according to the scheme previously described for the subunit vaccine based on the S1 antigen (Chen et al. 2024). A comparative analysis of the immunogenicity and protectiveness of the VLP-vaccine with an inactivated vaccine (CZ2020 strain) was performed on piglets born from vaccinated sows. No substantial difference was revealed after challenge with homologous CZ2020 strain (Zhang et al. 2024b).

In research performed by Li et al. in 2024, an mRNA-vaccine packed in lipid nanoparticles for target delivery and *in vivo* expression was generated. S-protein or S-protein ectodomain of PDCoV were used as vaccine antigens. At the first stage, the immunogenicity of both vaccine variants was tested on a mouse model. Although IgG and neutralising antibody titres did not significantly differ, an mRNA-vaccine based on full-size S-protein was selected for studies of protective efficacy evaluation. Experiments on passive protection assessment were conducted on suckling piglets, using inactivated vaccine for comparative evaluation. The mRNA-vaccine elicited higher IgA titres and, according to histopathological studies, protected five days old piglets against a CZ2020 strain challenge better than the inactivated vaccine (Li et al. 2024c).

## 5. Conclusion

The livestock and poultry industry worldwide is facing severe economic losses due to diseases caused by multiple variants and strains of coronaviruses affecting cattle, pigs and poultry. The spread of these viruses is determined by many factors, including geographic location, animal husbandry practices and disease prevention measures. Nevertheless, despite ongoing efforts, CoV remains widespread in farm animal populations all over the world (Table 1). CoV is characterised by the high genetic variability of strains and adaptation to the host due to frequent recombination and mutations, including point mutations, insertions and deletions during RNA replication. Prolonged latent infections facilitate these processes. The emergence of new epidemiologically significant CoV variants in farm animals may lead to unintended consequences, not only for livestock farming but also for public health. The zoonotic potential of CoV is quite high and the ability of interspecies transmission to humans has already been confirmed for BCoV and the fairly recently spread porcine deltacoronavirus.

Vaccination against viruses that cause acute and chronic infectious diseases is still the most effective method in the fight to reduce their spread and to subsequently eliminate them. Currently, vaccines that are currently widely-used worldwide against economically important farm animals, such as BCoV, IBV, TGEV and PEDV, are live-attenuated or inactivated vaccines (Table 1). The high variability of CoV and the emergence of new variants with high epidemiologic potential dramatically decrease the effectiveness of vaccination and require the constant updating of vaccine strains. Live-attenuated vaccine utilisation in the field involves a high safety risk. A reversion to the pathogenic strain may occur not only due to mutations but also as a result of recombination of the vaccine strain with circulating variants of wild-type strains. Such new recombinant strains of IBV and PEDV have been detected. This, in turn, can lead to vaccination failure and the loss of control over the epidemiological situation. Inactivated vaccines require virus strain cultivation and confirmation of inactivation completeness that involves certain risks. Inactivated vaccines are poorer immunogenic than live-attenuated vaccines, which implies the necessity for more intensive vaccination regimes and the application of adjuvants. Moreover, a parenteral route for administering inactivated vaccines is associated with a low level of effectiveness in stimulating a lactogenic immune response, which is important for the protection of neonatal piglets and calves, which constitute the main risk group, with high mortality rates (Table 1). The constant emergence of new CoVs with epidemic potential increases the importance of the development of innovative strategies for the design of safe and effective vaccines, and for ensuring a rapid response to arising challenges.

Experimental recombinant vaccines against economically important coronavirus infections of farm animals are designed using two main approaches: (1) through vaccines that provide expression target recombinant antigens *in vivo* in host cells (live bacterial vector vaccines, virus vector vaccines, DNA-vaccines, RNA-vaccines); (2) through vaccines that include recombinant proteins or VLPs, produced in various expression systems (Table 2). In numerous studies, mostly conducted in 2024, the effectiveness of various recombinant vaccines against PEDV, IBV, BCoV and PDCoV, including subunit vaccines, VLP-vaccines, virus vector vaccines, DNA vaccines and RNA-vaccines, has been compared with the effectiveness of live-attenuated and inactivated vaccines. Assessments of protective efficacy conducted through target animal challenge with a virulent strain and/or performing virus-neutralising tests on cell cultures have demonstrated that recombinant vaccines are as effective as whole-virus vaccines, and in some cases even superior to them. As with licensed vaccines, although recombinant vaccines’ ability to considerably decrease the clinical signs of infection and mortality has been demonstrated, they cannot completely prevent virus release into the environment. Although the efficacy of traditional and recombinant vaccines is similar, recombinant vaccines have a number of advantages. They are safe, available and easier to manufacture, and they can be updated in response to any newly emerging CoV variant. All of these properties make recombinant vaccines the preferred tool for controlling coronavirus infections, over live-attenuated and inactivated vaccines. Furthermore, researchers and developers in this field can optimise recombinant vaccines’ antigenic content, explore various methods of vaccine administration as part of combined and heterologous vaccination regimes as well as with application of new adjuvants. In conditions of high genetic variability of CoV, it is necessary to develop vaccines with a broader spectrum of action, capable of inducing a cross-protective immune response against different genotypes and strains of the virus, including new ones emerging as a result of mutations. A promising direction in the design of such vaccines are polyepitopic vaccines that include conservative and/or consensus antigenic determinants or fragments relevant to a given geographic region. In addition to classical immunological methods, bioinformatic analysis can be useful for predicting and characterizing antigenic epitopes and creating broad-spectrum vaccines. A number of studies have already developed polyepitope vaccines against farm CoV *in silico* (Table 2). To assess their immunogenicity and protectivity, it is necessary to conduct experimental studies on target animal species. This will not only confirm theoretical assumptions but also identify potential limitations and directions for further improvement of vaccines. The generation of new vaccines to protect farm animals from CoV is critical to restricting the spread of viruses and minimising the risks that CoV may pose to human health.

## Data Availability

The datasets analyzed for this review are available upon request from the corresponding author. Requests to access the datasets should be directed to Nikolai Nikitin at nikitin@mail.bio.msu.ru.
